# The Impact of Reduced Nitrogen Fertilizer Application and *Arbuscular mycorrhizal fungi* Inoculation on Nitrogen Utilization in Intercropped *Areca catechu* L. and *Vanilla planifolia* Andrews

**DOI:** 10.3390/plants14203207

**Published:** 2025-10-18

**Authors:** Huifa Zhuang, Xinyu Tang, Ziwei Ning, Chengjun Zhou, Qingyun Zhao, Hui Wang, Yizhang Xing, Ang Zhang

**Affiliations:** 1 Spice and Beverage Research Institute, Chinese Academy of Tropical Agricultural Sciences/Ministry of Agriculture and Rural Affairs Key Laboratory of Genetic Resource Utilization of Spice and Beverage Crops/Hainan Key Laboratory of Genetic Improvement and Quality Regulation for Tropical Spice and Beverage Crops, Wanning 571533, China; 2 Tropical Crops College, Yunnan Agricultural University, Puer 665000, China

**Keywords:** flavoring crop, mycorrhizal symbiosis, mixed cropping, nitrogen use efficiency

## Abstract

Areca (*Areca catechu* L.) is an important economic crop in tropical regions, but excessive nitrogen application leads to low nitrogen fertilizer utilization efficiency (approximately 30%). Vanilla (*Vanilla planifolia* Andrews) can be intercropped with areca to enhance land use efficiency. However, the impact of combined nitrogen reduction and *Arbuscular mycorrhizal fungi* (AMF) inoculation on the intercropping system of areca and vanilla remains unclear. This study examined the impact of nitrogen reduction (at levels of conventional fertilization, a 30% reduction and a 60% reduction) and the inoculation of AMF on the photosynthetic characteristics, physiological metabolism, and nitrogen utilization within an areca and vanilla intercropping system, employing a two-factor experimental design. The nitrogen reduction significantly inhibited SPAD value (chlorophyll content) (decreased by 46.21%), net photosynthesis (P_n_) (decreased by 71.13%), and transpiration rate (T_r_) (decreased by 44.34%) of vanilla without inoculation of AMF, but had little effect on the photosynthesis of areca. Inoculation with AMF, notably *Funneliformis mosseae*, alleviated the adverse effects of reduced nitrogen on vanilla. The net photosynthesis and intercellular CO_2_ concentration (C_i_) significantly increased by 76.23% and 69.48%, respectively. Additionally, the nitrogen uptake efficiency of the areca was improved, with root vitality increasing by 39.96%. Additionally, AMF enhanced the activities of acid phosphatase (ACP) (increased by 38.86% in vanilla) and nitrate reductase (NR) (increased by 53.77% in areca), promoting soil mineral nutrient activation and nitrogen metabolism. The nitrogen reduction combined with AMF inoculation can improve the nitrogen use efficiency of the areca and vanilla intercropping system, revealing its synergistic mechanism in the tropical intercropping system.

## 1. Introduction

Areca (*Areca catechu* L.) is a perennial evergreen tree of the Arecaceae family, preferring high temperature and humidity, and is a typical tropical plant [1]. Areca is not only a typical tropical landscape tree species but also an important medicinal plant, listed as one of the four major southern medicinal plants [2]. Its fruit, seeds, and flowers can all be used for medicinal purposes. The planting area of areca in Hainan Province is 173,100 hectares, with a total output of 276,000 tons. It is the second-largest economic crop in Hainan, next to rubber, and is also an important source of income for farmers in the tropical regions. With the continuous increase in the price of areca, the application of nitrogen fertilizer has also been increasing. However, studies have shown that the yield of areca has not increased with the increase in nitrogen application but has remained at a relatively stable level. The nitrogen fertilizer utilization rate in areca plantations is only about 30%, and excessive nitrogen application has led to increasingly serious groundwater nitrate pollution [3].

Vanilla (*Vanilla planifolia* Andrews) is a perennial tropical climbing plant of the Orchidaceae vanilla, known as the “King of Natural Food Flavors” [4]. The pods contain over 250 volatile aromatic components after flavoring processing and are one of the world’s most valuable spice plants [5]. They are mainly used for high-end food and cosmetic flavoring and have health benefits such as kidney tonification, stomach strengthening, bloating relief, and detoxification of the liver [6]. Vanilla features well-established shallow aerial roots and is well-suited to shaded conditions [7]. Investigations have revealed that growing vanilla in the semi-shaded settings of areca nut farms can substantially boost yield per land area. Furthermore, the interplay of roots can considerably enhance the plants’ ability to absorb nitrogen, their efficiency in utilizing nitrogen, and their rate of nitrogen fertilizer use.

*Arbuscular mycorrhizal fungi* (AMF) are an important part of the soil microbial community and can establish good symbiotic relationships with most plants [8]. Applying chemical fertilizers to promote AMF symbiosis to help shallow-rooted plants obtain more nutrients is of great significance in improving the current over-fertilization in crop production [9]. It can not only effectively improve soil compaction but also enhance soil water and fertilizer retention, reduce soil pathogenic fungi, and promote the growth of beneficial fungi [10]. Studies have found that under the same nitrogen application treatment, intercropping significantly increased the biomass and nitrogen uptake of areca and vanilla, but the total nitrogen content in the soil did not decrease due to biological nitrogen fixation.

This study monitored the effects of reduced nitrogen application and inoculation with *Arbuscular mycorrhizal fungi* (AMF) on the photosynthetic characteristics, physiological metabolism, and nitrogen use efficiency of two crops. It aimed to determine whether inoculating with AMF could alleviate the inhibitory effects of reduced nitrogen application on the photosynthesis and metabolism of vanilla, and whether there was an interaction between reduced nitrogen application and AMF inoculation on the nitrogen use efficiency of the two crops. The result of this study will provide a theoretical basis for reducing chemical fertilizers and improving fruit quality and efficiency in intercropping gardens.

## 2. Results

### 2.1. Effects of Nitrogen Fertilizer Reduction and AMF Inoculation on the Photosynthetic Characteristics of Vanilla and Areca

Nitrogen application rates and AMF inoculation treatments significantly affected SPAD values, net photosynthetic rate (Pn), and transpiration rate (Tr) in vanilla, as well as intercellular CO_2_ concentration (Ci) and stomatal conductance (Gs) in betel nut (*p* < 0.05). Different capital letters in Table 1 and Table 2 (A, B, C) indicate significant differences (*p* < 0.05) among inoculation treatments at the same nitrogen application level, visually reflecting the aforementioned significant interactions. A comparison with conventional fertilization revealed that nitrogen reduction notably suppressed SPAD value, Pn, and Tr of vanilla under non-inoculated control (NAM) treatment (Table 1). Under conventional fertilization (C) levels, the SPAD values of vanilla plants inoculated with AMF (F.m) were significantly lower than those of the non-inoculated treatment, indicating that inoculation with this fungal strain actually reduced chlorophyll content at this nitrogen level. However, a 30% reduction in nitrogen resulted in an increase in stomatal conductance (G_s_), whereas a 60% reduction in nitrogen did not significantly affect G_s_ or C_i_ compared with conventional fertilization. When contrasted with conventional fertilization (C), SPAD value, P_n_, and T_r_ of vanilla experienced decreases of 46.21%, 71.13%, and 44.34%, respectively. Conversely, G_s_ saw a 100% increase under 30% nitrogen reduction. Under the treatment of *Claroideoglomus etunicatum* (AMF (C.e)), compared with C, the reduction in fertilization promoted the P_n_ of vanilla, and had no significant effect on T_r_ and SPAD value. Compared with C, the 60% reduction in nitrogen significantly decreased C_i_ and G_s_ of vanilla. The 30% reduction in nitrogen had no effect on C_i_ and G_s_. Compared with C, a 60% reduction in fertilizer application resulted in respective increases of 76.23%, 69.48%, and 88.89% in the P_n_, C_i_, and G_s_.

In the group inoculated with *Funneliformis mosseae* (AMF (F.m)), compared with conventional fertilization, 30% reduced fertilization promoted P_n_ and T_r_, and 60% reduction in fertilization promoted C_i_ and G_s_, with increases of 12.25%, 51.79%, 24.35%, and 114.29%, respectively. The 60% reduction in fertilization led to a 14.1% reduction in SPAD value, while a 30% reduction in fertilization did not significantly affect G_s_.

In the non-inoculated control group, compared with conventional fertilization, the reduction in fertilizer application compared to the conventional fertilization had an inhibitory effect on SPAD value, P_n_, and C_i_ of the areca, and had a significant effect on G_s_. A 30% reduction in fertilizer application had no significant effect on T_r_, while a 6% reduction in fertilizer application had an inhibitory effect on T_r_. Compared to conventional fertilization, the SPAD value, P_n_, and C_i_ of the areca were reduced by 45.72%, 80.70%, and 88.79%, respectively, and T_r_ was reduced by 27.28% (Table 2).

In the group inoculated with AMF (C.e), the reduction in fertilizer application compared with conventional fertilization had a promoting effect on P_n_ and C_i_ of areca, increasing by 86.99% and 16.17%, respectively. The reduction of 30% fertilizer application had no significant effect on T_r_, and the reduction of 60% application increased G_s_ by 90%, and the reduction of 60% fertilizer application had a promoting effect on G_s_, the reduction of 60% fertilizer application had an inhibitory effect on T_r_, and Tr under the reduction of 60% fertilizer application decreased by 73.68%. In the group inoculated with AMF (F.m), compared with conventional fertilization, reduced fertilization promoted C_i_ and G_s_, which increased by 82.26% and 85%, respectively; a 30% reduction in fertilization had an inhibitory effect on P_n_ and T_r_, which decreased by 16.35% and 43.4%, respectively; when the fertilizer was reduced by 60%, the SPAD value decreased by 24.14%, and the effect on P_n_ was not significant (Table 2).

### 2.2. The Effects of Nitrogen Fertilizer Reduction and AMF Inoculation on the Soluble Sugar Content of Plants

The results of two-factor variance analysis showed that the fertilization and inoculation treatment had a significant effect on the soluble sugar content of vanilla and areca (all *p* < 0.0001), and the two treatments had an interaction effect on soluble sugar content of vanilla and areca in this study (Table 3). Under conventional fertilization (C) conditions, the soluble sugar content of non-inoculated betel nut (NAM) was significantly higher than that of the two AMF-inoculated treatments. This indicates that when nitrogen is abundant, inoculating with AMF actually inhibits sugar accumulation in betel nut. Under 60% nitrogen reduction, no significant differences in soluble sugar content were observed among the three inoculated treatments, suggesting that the effects of inoculation were diminished under severe nitrogen limitation. The soluble sugar content of vanilla was higher under 30% reduced fertilization than under conventional fertilization and 60% reduced fertilization, and the soluble sugar content of areca was significantly higher under fertilization than under 30% and 60% reduced fertilization; the soluble sugar content of vanilla and areca in the uninoculated control was higher than that in AMF (C.e) and AMF (F.m) (Table 3).

**Figure 1:**
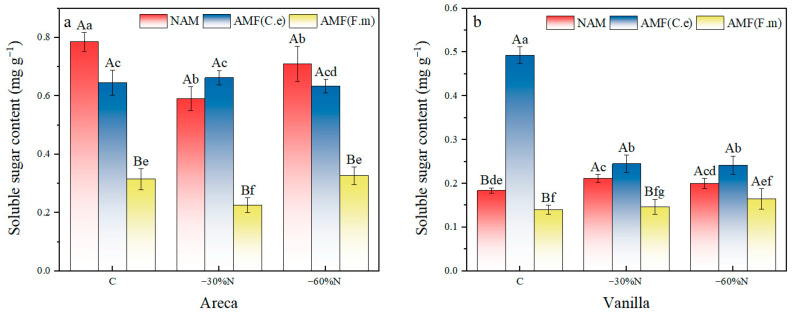
Influence of coordinated regulation of nitrogen fertilizer reduction and AMF inoculation on the soluble sugar content. (**a**) Areca; (**b**) Vanilla.NAM, non-inoculated control; (AMF (C.e)), inoculated with *Claroideoglomus etunicatum*; (AMF (F.m)), inoculated with *Funneliformis mosseae*; C, conventional fertilization; −30%N, 30% reduction; −60%N, 60% reduction. Data are presented as the mean ± standard error (n = 5). Values labeled with different lowercase letters differ significantly at the 5% significance level (*p* < 0.05) under the same inoculation condition but different fertilization treatments. Different uppercase letters indicate significant differences at the 5% significance level (*p* < 0.05) under the same fertilization condition but different inoculation treatments.

In the non-inoculated control group, both 30% nitrogen reduction and 60% nitrogen reduction significantly decreased the soluble sugar content of areca compared with conventional fertilization, with reductions of 24.71% and 9.55%, respectively (Figure 1). Similarly, the soluble sugar content of vanilla was also decreased by 15.22% and 8.70% under 30% and 60% nitrogen reduction compared with conventional fertilization. However, the effect of 60% nitrogen reduction had no effect on vanilla. In the group inoculated with AMF (C.e), 30% nitrogen reduction increased the soluble sugar content of areca by 2.64%, while 60% nitrogen reduction decreased it by 1.86% compared with conventional fertilization. The effect of nitrogen reduction had no effect on the soluble sugar content of areca. For vanilla, both 30% and 60% nitrogen reduction decreased the soluble sugar content by 50.30% and 50.91%, respectively, compared with conventional fertilization. In the group inoculated with AMF (F.m), 30% nitrogen reduction decreased the soluble sugar content of areca by 47.62%, while 60% nitrogen reduction increased it by 3.81%, compared with conventional fertilization. However, this effect was not significant. For vanilla, both 30% and 60% nitrogen reduction decreased the soluble sugar content by 5.00% and 17.86%, respectively, compared with conventional fertilization. The effect of nitrogen reduction had no effect on the soluble sugar content of vanilla.

In summary, in NAM, 60% nitrogen reduction had no effect on the soluble sugar content of vanilla. In the group inoculated with AMF (C.e), the effect of nitrogen reduction on the soluble sugar content of areca was not significant. In the group inoculated with AMF (F.m), the effect of 60% nitrogen reduction on the soluble sugar content of areca was not significant, and nitrogen reduction had no effect on the soluble sugar content of vanilla.

### 2.3. The Effect of Reduced Nitrogen Fertilizer and AMF Inoculation on the Content of Soluble Protein in Plants

The results of two-factor variance analysis showed that inoculation treatment had a significant effect on the soluble protein content of vanilla (*p* <0.05). There is a significant interaction between the effects of fertilization and inoculation on the soluble protein content of areca (*p* < 0.01, Table 4). Taking betel nut as an example, under conventional fertilization (C), the protein content in treatments inoculated with AMF (C.e) was significantly higher than in non-inoculated treatments, demonstrating the positive effect of AMF under conventional nitrogen application. However, when nitrogen was reduced by 30% (−30% N), no significant differences were observed among all inoculated treatments, suggesting that moderate nitrogen reduction may have balanced the effects of different inoculation methods. The soluble protein content of vanilla fertilized with 30% and 60% less fertilizer was significantly higher than that of conventional fertilization (60%).

**Figure 2:**
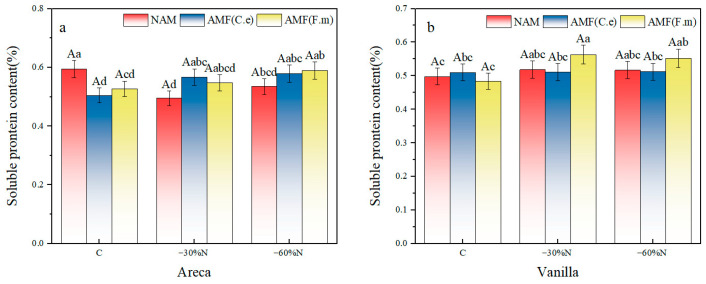
Influence of coordinated regulation of nitrogen fertilizer reduction and AMF inoculation on the soluble protein content. (**a**) Areca; (**b**) Vanilla. NAM, non-inoculated control; (AMF (C.e)), inoculated with *Claroideoglomus etunicatum*; (AMF (F.m)), inoculated with *Funneliformis mosseae*; C, conventional fertilization; −30%N, 30% reduction; −60%N, 60% reduction. Data are presented as the mean ± standard error (n = 5). Values labeled with different lowercase letters differ significantly at the 5% significance level (*p* < 0.05) under the same inoculation condition but different fertilization treatments. Different uppercase letters indicate significant differences at the 5% significance level (*p* < 0.05) under the same fertilization condition but different inoculation treatments.

In the non-inoculated control group, the soluble protein content of areca decreased by 16.81% and 1.08% under 30% and 60% fertilization reduction compared with the conventional fertilization; the soluble protein content of vanilla increased by 4.02% and 3.82%, and the reduction in fertilization had no significant effect on the soluble protein content of vanilla (Figure 2). In the group inoculated with AMF (C.e), both 30% and 60% nitrogen reduction significantly increased the soluble protein content of areca by 12.28% and 14.65%, respectively, compared with conventional fertilization. Similarly, for vanilla, both 30% and 60% nitrogen reduction increased the soluble protein content by 0.2% and 0.39%, respectively, compared with conventional fertilization. However, nitrogen reduction had no effect on the soluble protein content of vanilla. In the group inoculated with AMF (F.m), both 30% and 60% nitrogen reduction increased the soluble protein content of areca by 3.80% and 11.95%, respectively, compared with conventional fertilization. However, a 30% nitrogen reduction had no effect on the soluble protein content of areca. For vanilla, both 30% and 60% nitrogen reduction increased the soluble protein content by 16.32% and 14.05%, respectively, compared with conventional fertilization. In summary, among the three inoculation treatments, in the group inoculated with AMF (F.m), 30% nitrogen reduction had no effect on the soluble protein content of areca. In NAM and the group inoculated with AMF (C.e), nitrogen reduction had no effect on the soluble protein content of vanilla.

### 2.4. The Effect of Reduced Nitrogen Fertilizer and AMF Inoculation on the Activity of Glutamine Synthetase in Plants

**Figure 3:**
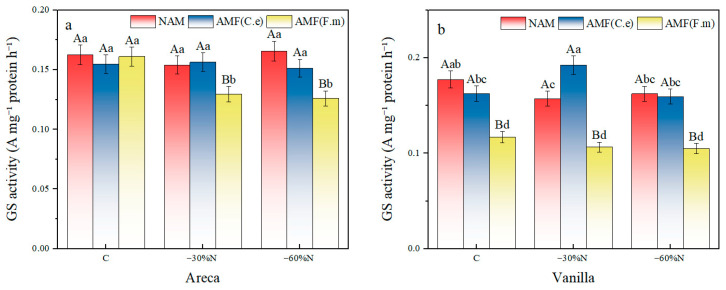
Influence of coordinated regulation of nitrogen fertilizer reduction and AMF inoculation on the glutamine synthetase activity. (**a**) Areca; (**b**) Vanilla. NAM, non-inoculated control; (AMF (C.e)), inoculated with *Claroideoglomus etunicatum*; (AMF (F.m)), inoculated with *Funneliformis mosseae*; C, conventional fertilization; −30%N, 30% reduction; −60%N, 60% reduction. Data are presented as the mean ± standard error (n = 5). Values labeled with different lowercase letters differ significantly at the 5% significance level (*p* < 0.05) under the same inoculation condition but different fertilization treatments. Different uppercase letters indicate significant differences at the 5% significance level (*p* < 0.05) under the same fertilization condition but different inoculation treatments.

The results of two-factor variance analysis showed that the fertilization treatment had a significant effect on the glutamine synthetase (GS) activity of vanilla (*p* < 0.05), but not on the GS activity of areca. There was no interaction between fertilization and inoculation on the GS activity of vanilla and areca (Table 5). Consistent with this analysis, the capital letters marking the majority of items within each treatment group in Figure 3 are largely identical.

Under the non-inoculated control group (NAM), the GS content of Areca catechu decreased by 5.17% in the 30% reduction and increased by 1.97% in the 60% reduction compared with the fertilization, and the reduction in fertilization had no significant effect on the GS activity of areca; compared with the conventional fertilization, both the 30% and 60% reduction fertilization decreased the GS content of vanilla by 11.29% and 8.45%, respectively (Figure 3). In the group inoculated with AMF (C.e), compared with conventional fertilization, both 30% and 60% nitrogen reduction increased the GS content in areca by 0.90% and 2.33%, respectively. Nitrogen reduction had no effect on GS activity in areca. For vanilla, a 30% nitrogen reduction increased the GS content by 18.34%, while a 60% nitrogen reduction decreased it by 1.85%, compared with conventional fertilization. A 60% nitrogen reduction had no effect on GS activity in vanilla. In the group inoculated with AMF (F.m), compared with conventional fertilization, both 30% and 60% nitrogen reduction decreased the GS activity in areca by 19.63% and 21.74%, respectively. For vanilla, both 30% and 60% nitrogen reduction decreased the GS content by 8.9% and 10.10% compared with conventional fertilization. Nitrogen reduction had no effect on GS activity in vanilla.

In summary, among the three inoculation treatments in NAM, nitrogen reduction had no effect on GS activity in areca. In the group inoculated with AMF (C.e), nitrogen reduction had no effect on GS activity in areca, and 60% nitrogen reduction had no effect on GS activity in vanilla. In the group inoculated with AMF (F.m), nitrogen reduction had no effect on GS activity in vanilla.

### 2.5. The Effect of Synergistic Regulation of Reduced Nitrogen Fertilizer and AMF Inoculation on the Vitality of Plant Root System

The results of two-factor variance analysis showed that inoculation treatment had a significant effect on vanilla root activity (*p* < 0.0001), among which the vanilla root activity of inoculation AMF (F.m) was significantly higher than that of inoculation NAM treatment by 30.6%. The fertilization and inoculation had a significant interaction effect on the root activity of areca (*p* < 0.05) (Table 6). At all nitrogen application rates for betel nut, root vitality in treatments inoculated with AMF (F.m) was significantly higher than in the non-inoculated control (NAM). For vanilla, at the −60% N rate, root vitality in the AMF (F.m)-inoculated treatment was significantly higher than in both the non-inoculated control and the AMF (C.e)-inoculated treatment.

**Figure 4:**
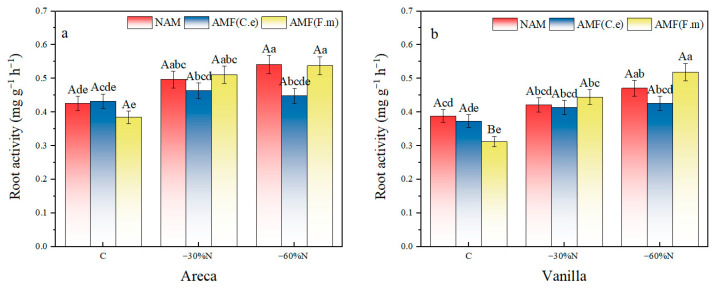
Influence of coordinated regulation of nitrogen fertilizer reduction and AMF inoculation on the root activity. (**a**) Areca; (**b**) Vanilla. NAM, non-inoculated control; (AMF (C.e)), inoculated with *Claroideoglomus etunicatum*; (AMF (F.m)), inoculated with *Funneliformis mosseae*; C, conventional fertilization; −30%N, 30% reduction; −60%N, 60% reduction. Data are presented as the mean ± standard error (n = 5). Values labeled with different lowercase letters differ significantly at the 5% significance level (*p* < 0.05) under the same inoculation condition but different fertilization treatments. Different uppercase letters indicate significant differences at the 5% significance level (*p* < 0.05) under the same fertilization condition but different inoculation treatments.

Both 30% nitrogen reduction and 60% nitrogen reduction significantly increased root activity in areca compared with conventional fertilization, with increases of 16.78% and 27.11% under NAM treatment (Figure 4). Similarly, for vanilla, both 30% and 60% nitrogen reduction increased root activity by 8.54% and 21.26% compared with conventional fertilization. However, 30% nitrogen reduction had no effect on root activity in vanilla. In the group inoculated with AMF (C.e), both 30% and 60% nitrogen reduction increased root activity in areca by 7.22% and 3.75%, respectively, compared with conventional fertilization. Nitrogen reduction had no effect on root activity in areca. For vanilla, both 30% and 60% nitrogen reduction increased root activity by 10.76% and 13.87%, respectively, compared with conventional fertilization. Nitrogen reduction had no effect on root activity in vanilla. In the group inoculated with AMF (F.m), both 30% and 60% nitrogen reduction significantly increased root activity in areca by 33.04% and 39.96%, respectively, compared with conventional fertilization. For vanilla, both 30% and 60% nitrogen reduction increased root activity by 42.38% and 66.13%, respectively, compared with conventional fertilization.

In summary, in NAM, 30% nitrogen reduction had no effect on root activity in vanilla. In the group inoculated with AMF (C.e), nitrogen reduction had no effect on root activity in both areca and vanilla.

### 2.6. The Effect of Reduced Nitrogen Fertilizer and AMF Inoculation on the Activity of Nitrate Reductase in Plants

The results of two-factor variance analysis showed that the interactive effect of fertilization and inoculation had no significant effect on the nitrate reductase (NR) activity of vanilla. There is no interaction between the effects of fertilization and inoculation on the NR activity of areca (all *p* > 0.1), but there was a significant interaction effect on the NR activity of vanilla (*p* < 0.05) (Table 7). For vanilla orchids, under 60% nitrogen reduction (−60%N) conditions, the NR activity of the AMF (F.m)—inoculated treatment was significantly higher than that of the non-inoculated treatment. This indicates that inoculation with AMF (F.m) effectively enhances the nitrate reduction capacity of vanilla orchids under low nitrogen stress. In contrast, under all nitrogen application levels for betel nut, no significant differences were observed among inoculation treatments. This aligns with the statistically insignificant interaction results, indicating that inoculation with AMF has limited effects on betel nut’s NR activity.

**Figure 5:**
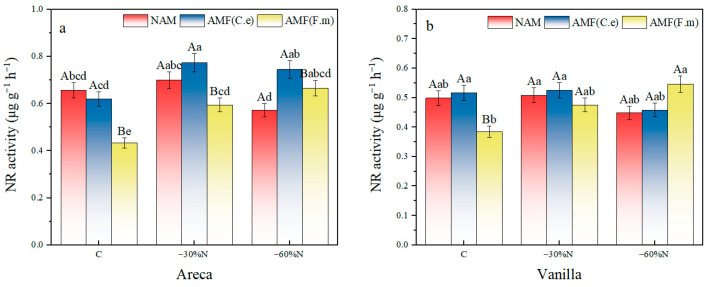
Influence of coordinated regulation of nitrogen fertilizer reduction and AMF inoculation on the nitrate reductase activity. (**a**) Areca; (**b**) Vanilla. NAM, non-inoculated control; (AMF (C.e)), inoculated with *Claroideoglomus etunicatum*; (AMF (F.m)), inoculated with *Funneliformis mosseae*; C, conventional fertilization; −30%N, 30% reduction; −60%N, 60% reduction. Data are presented as the mean ± standard error (n = 5). Values labeled with different lowercase letters differ significantly at the 5% significance level (*p* < 0.05) under the same inoculation condition but different fertilization treatments. Different uppercase letters indicate significant differences at the 5% significance level (*p* < 0.05) under the same fertilization condition but different inoculation treatments.

Compared with conventional fertilization, 30% nitrogen reduction increased the nitrate reductase content in areca by 6.59%, while 60% nitrogen reduction decreased it by 12.84% under NAM treatment (Figure 5). Nitrogen reduction had no effect on nitrate reductase activity. For vanilla, 30% nitrogen reduction increased the nitrate reductase content by 1.97%, while 60% nitrogen reduction decreased it by 9.83%, compared with conventional fertilization. Nitrogen reduction had no effect on nitrate reductase activity.

In the group inoculated with AMF (C.e), compared with conventional fertilization, both 30% and 60% nitrogen reduction increased the nitrate reductase content in areca by 24.86% and 20.18%, respectively. For vanilla, a 30% nitrogen reduction increased the nitrate reductase content by 1.67%, while a 60% nitrogen reduction decreased it by 11.31%, compared with conventional fertilization. Nitrogen reduction had no effect on nitrate reductase activity in vanilla. In the group inoculated with AMF (F.m), compared with conventional fertilization, both 30% and 60% nitrogen reduction increased the nitrate reductase content in areca by 37.36% and 53.77%, respectively (Figure 5). For vanilla, both 30% and 60% nitrogen reduction increased the nitrate reductase content by 23.57% and 40.09%, respectively, compared with conventional fertilization. 30% nitrogen reduction had no effect on nitrate reductase activity in vanilla.

In summary, in NAM, nitrogen reduction had no effect on nitrate reductase activity in either areca or vanilla. In the group inoculated with AMF (C.e), nitrogen reduction had no effect on nitrate reductase activity in vanilla. In the group inoculated with AMF (F.m), 30% nitrogen reduction had no effect on nitrate reductase activity in vanilla.

### 2.7. The Effects of Reduced Nitrogen Fertilizer and AMF Inoculation on the Activity of Acid Phosphatase in Plants

The results of the two-factor variance analysis showed that fertilization and inoculation had a significant effect on the acid phosphatase (ACP) activity of vanilla (*p* < 0.1), and that fertilization had a significant effect on the ACP activity of areca (*p* < 0.05), among which the ACP activity of orchid treated with conventional fertilization was significantly lower than that of 30% and 60% reduction fertilization, which was 21.43% and 18.60% lower, respectively; the ACP activity of vanilla treated with AMF (C.e) and AMF (F.m) was significantly higher than that of NAM treatment by 22.86% and 17.14%, respectively (Table 8). For vanilla orchids, under conventional fertilization (C) conditions, both AMF inoculation treatments exhibited significantly higher ACP activity than the non-inoculated treatment, demonstrating the positive effect of AMF inoculation on activating soil phosphorus at conventional nitrogen levels. The disappearance of differences among inoculation treatments under reduced nitrogen conditions suggests that nitrogen reduction may become the dominant factor, diminishing the effect of differences between inoculation treatments.

**Figure 6:**
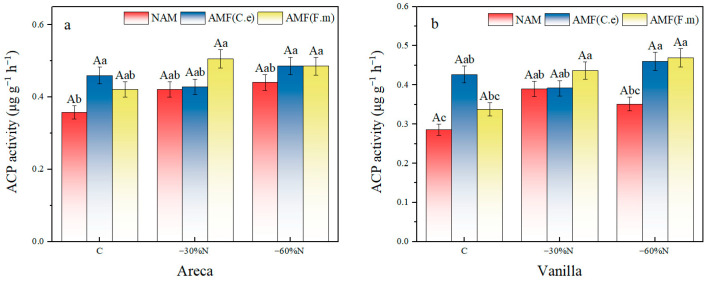
Influence of coordinated regulation of nitrogen fertilizer reduction and AMF inoculation on the acid phosphatase activity. (**a**) Areca; (**b**) Vanilla. NAM, non-inoculated control; (AMF (C.e)), inoculated with *Claroideoglomus etunicatum*; (AMF (F.m)), inoculated with *Funneliformis mosseae*; C, conventional fertilization; −30%N, 30% reduction; −60%N, 60% reduction. Data are presented as the mean ± standard error (n = 5). Values labeled with different lowercase letters differ significantly at the 5% significance level (*p* < 0.05) under the same inoculation condition but different fertilization treatments. Different uppercase letters indicate significant differences at the 5% significance level (*p* < 0.05) under the same fertilization condition but different inoculation treatments.

Compared with conventional fertilization, both 30% and 60% nitrogen reduction increased the content of acid phosphatase in areca by 17.74% and 23.11% under NAM treatment (Figure 6). Nitrogen reduction had no effect on acid phosphatase activity in areca. For vanilla, both 30% and 60% nitrogen reduction increased the content of acid phosphatase by 36.46% and 22.95%. Compared with conventional fertilization. A 60% nitrogen reduction had no effect on acid phosphatase activity in vanilla.

In the group inoculated with AMF (C.e), compared with conventional fertilization, 30% nitrogen reduction decreased the content of acid phosphatase in areca by 6.79%, while 60% nitrogen reduction increased it by 5.79%. The effect of nitrogen reduction on acid phosphatase activity in areca was not significant. For vanilla, 30% nitrogen reduction decreased the content of acid phosphatase by 8.2%, while 60% nitrogen reduction increased it by 7.87%, compared with conventional fertilization. Nitrogen reduction had no effect on acid phosphatase activity in vanilla. In the group inoculated with AMF (F.m), compared with conventional fertilization, both 30% and 60% nitrogen reduction increased the content of acid phosphatase in areca by 20.20% and 15.30%, respectively. Nitrogen reduction had no effect on acid phosphatase activity in areca. For vanilla, both 30% and 60% nitrogen reduction increased the content of acid phosphatase by 29.37% and 38.86%, respectively, compared with conventional fertilization.

In summary, in all three inoculation treatments, nitrogen reduction had no effect on acid phosphatase activity in vanilla. In NAM, the effect of 60% nitrogen reduction on acid phosphatase activity in vanilla was not significant. In the group inoculated with AMF (C.e), nitrogen reduction had no effect on acid phosphatase activity in vanilla.

### 2.8. Under the Coordinated Regulation of Reduced Nitrogen Fertilization and AMF Inoculation, the Relationship Between Net Photosynthetic Rate and Soluble Protein Content and Root Activity Has Been Established

**Figure 7:**
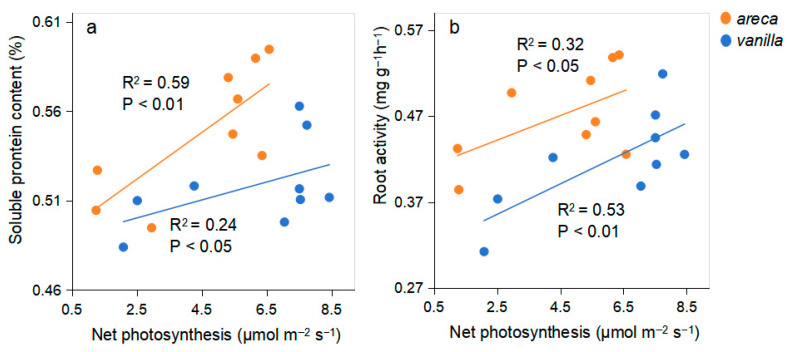
The relationship between net photosynthesis and plant physiological characteristics in areca and vanilla. (**a**) Areca; (**b**) Vanilla.

Soluble protein content and root activity are positively correlated with net photosynthesis in both species, albeit with varying degrees of strength (Figure 7). Areca shows a stronger relationship with soluble protein content, while vanilla exhibits a stronger relationship with root activity. These findings imply that increased photosynthetic rates may be associated with higher soluble protein levels in areca and enhanced root activity in vanilla.

## 3. Discussion

Nitrogen, through its involvement in chlorophyll synthesis, the construction of the photosynthetic enzyme system, and the transport of photosynthetic products, has become one of the key factors regulating the intensity of plant photosynthesis [11]. A moderate supply of nitrogen can enhance photosynthetic efficiency, while nitrogen deficiency can lead to dysfunction of the photosynthetic apparatus [12]. In this study, the effects of reduced nitrogen fertilization on the photosynthetic characteristics of areca and vanilla were different. This may be related to the higher dependence of vanilla on nitrogen [13]. However, the areca can maintain high photosynthetic efficiency under low nitrogen fertilizer conditions, which may be related to the fact that the areca has developed a fiber root system and stronger nutrient acquisition ability. At the same time, this also shows that the areca may not be sensitive to the reduction of nitrogen fertilizer. In sustainable agricultural research, inoculation with AMF can enhance plant photosynthetic efficiency by increasing nutrient uptake, especially phosphorus and nitrogen [14,15]. In this study, inoculation with AMF significantly increased the net photosynthetic rate and intercellular CO_2_ concentration of vanilla plants under nitrogen-limited conditions. This demonstrates a pronounced interactive effect: the beneficial role of AMF is maximized under nitrogen stress conditions. This indicates that AMF can improve photosynthetic efficiency by promoting nutrient absorption, enhancing the function of the photosynthetic apparatus, and increasing stress resistance, thereby achieving coordinated improvement in nitrogen use efficiency and photosynthetic productivity [16]. Furthermore, two-way analysis revealed that AMF inoculation also enhanced the root vitality of areca plants, exhibiting a significant synergistic effect when combined with reduced nitrogen treatment (interaction *p* < 0.05). This indicates that AMF colonization improved root development and nutrient uptake efficiency, particularly under low-nitrogen conditions, thereby mitigating the adverse effects of nitrogen deficiency. Therefore, AMF inoculation can improve nitrogen uptake efficiency and mitigate the negative effects of reduced nitrogen fertilization on photosynthesis, which is consistent with previous studies [17].

Nitrogen directly affects the production of photosynthetic products by regulating chlorophyll synthesis, photosynthetic enzyme activity, and ATP supply, while also optimizing the distribution of photosynthetic products through phloem development and source-sink relationships [18,19]. In this study, reduced nitrogen fertilization had a minor effect on the soluble sugar and protein content of vanilla, but significantly decreased the soluble sugar and protein content of areca. Results indicate that the soluble sugar content of both plants was significantly influenced by the interaction between nitrogen fertilizer and AMF inoculation (*p* < 0.001), suggesting that carbon metabolism is highly sensitive to responses in synergistic regulation. This difference may be related to the varying sensitivity of the different crops to nitrogen levels [20]. The positive correlation between leaf photosynthetic rate and soluble protein content observed in this study suggests that some soluble proteins are core components of key photosynthetic enzymes. Nitrogen supply maintains the function of the photosynthetic apparatus by regulating protein synthesis [21].

Nitrogen enhances root activity by promoting root growth, increasing enzyme activity, and optimizing nutrient uptake [22]. However, reduced nitrogen fertilization in this study significantly enhanced root activity in both areca and vanilla. For areca, reduced nitrogen combined with *Arbuscular mycorrhizal fungi* (AMF) inoculation exhibited a significant synergistic effect (interaction *p* < 0.05). This indicates that either reduced nitrogen alone or AMF inoculation alone provides only limited improvement in root vitality, whereas the combination of both significantly amplifies the effect. This may be because reducing nitrogen application can enhance crop root activity through multiple pathways, including balancing root-top-ratio, optimizing nutrient uptake, regulating plant hormones, and improving the rhizosphere environment [23]. Additionally, leaf photosynthetic rate and root activity are closely linked through material exchange, hormone signaling, and energy metabolism [24]. Roots support photosynthesis by absorbing water and mineral nutrients and synthesizing hormones (e.g., CTK that promote stomatal opening) [25]. The positive correlation observed between the two variables in this study provides physiological evidence supporting the feasibility of nitrogen fertilizer and AMF synergistically regulating plant physiology through root–canopy signaling interactions.

Previous studies have shown that changes in soil nitrogen content can directly regulate NR activity to promote nitrogen assimilation, while also indirectly affecting acid phosphatase activity by altering soil pH and phosphorus availability [26]. Inoculation with AMF can significantly enhance plant nitrate reductase activity to strengthen nitrogen metabolism, while also increasing phosphorus uptake by boosting acid phosphatase activity [27]. Although the response of NR activity to synergistic regulation varied among crops (significant interaction was observed in vanilla, but not in areca), the increased acid phosphatase activity in vanilla plants indicates that mycorrhizal fungi help mitigate phosphorus deficiency caused by nitrogen limitation, thereby supporting growth under low-nitrogen conditions. Two-factor analysis confirmed that ACP activity was significantly influenced by nitrogen fertilizer and AMF inoculation (main effects *p* < 0.05), supporting the “substitution” strategy whereby AMF indirectly supports nitrogen metabolism by activating phosphorus nutrition under low-nitrogen conditions. Although nitrate reductase activity is susceptible to environmental conditions and plant species, the inconsistent responses of NR and acid phosphatase activity to nitrogen-reduced fertilization and AMF inoculation in this study precisely illustrate the complexity of the “nitrogen fertilizer-AMF” synergistic regulation. Its action may primarily target alleviating phosphorus limitation rather than directly enhancing nitrogen assimilation, indicating that AMF can improve nutrient use efficiency by regulating nitrogen and phosphorus metabolism in plants. This is consistent with previous findings that AMF can increase the activity of acid phosphatases secreted by plant roots, thereby promoting the release of insoluble phosphorus in the soil [28].

The results of this study, combined with two-way ANOVA, clearly indicate that nitrogen reduction and AMF inoculation do not exhibit a simple additive effect but rather a profound synergistic interaction. Nitrogen reduction combined with AMF inoculation can improve the nitrogen use efficiency of the areca and vanilla intercropping system through multiple pathways. AMF not only specifically alleviates the adverse effects of nitrogen reduction on vanilla photosynthesis but also synergistically enhances the nitrogen uptake efficiency of areca with nitrogen reduction. It further boosts soil nitrogen activation and the activity of key enzymes in plant nitrogen metabolism. This synergistic effect of nitrogen reduction and AMF inoculation provides a feasible technical approach for reducing nitrogen fertilizer application, improving nitrogen use efficiency, and promoting the sustainable development of the areca and vanilla intercropping system in tropical regions and a solid theoretical foundation.

This study first confirms the synergistic effect of nitrogen reduction and AMF inoculation in the areca–vanilla intercropping system. However, this study only explored the short-term effects of reduced nitrogen fertilization and AMF inoculation on the photosynthetic characteristics and soil enzyme activities of areca and vanilla. Future research will further investigate the long-term effects of different AMF species on the intercropping system of areca and vanilla, as well as the synergistic effects of AMF with other microorganisms.

## 4. Materials and Methods

### 4.1. Materials

The test crop varieties were Reyan No.1 areca and Reyin No.3 vanilla. Reyan No.1 areca was bred by the Coconut Research Institute of Chinese Academy of Tropical Agricultural Sciences, which had the characteristics of early fruiting, high yield, and good quality. Reyin No.3 vanilla was bred by the Institute of Perfume and Beverage, Chinese Academy of Tropical Agricultural Sciences. The test AMFs were *Claroideoglomus etunicatum* and *Funneliformis mosseae*, provided by the Institute of Plant Nutrition and Resources, Beijing Academy of Agriculture and Forestry Sciences. They were propagated for three months using sterile substrate and white clover (*Trifolium repens*) to produce the inoculum. Each inoculum sample contained approximately 50 spores per gram. Fertilizer was produced by Jiangsu Siweibo Biotechnology Co., Ltd. Using an advanced trough-type aerobic fermentation process to treat cattle manure. The specific process involves blending fresh cattle manure with auxiliary materials such as straw and mushroom residue in specific proportions to adjust the carbon-to-nitrogen ratio and porosity. After inoculation with a proprietary composite microbial agent (rich in thermophilic actinomycetes, Bacillus species, etc.), the mixture is placed in fermentation tanks to produce organic fertilizer. The finished product contains the following primary components: organic matter ≥ 45% and total nutrients (N + P_2_O_5_ + K_2_O) ≥ 5%. The test soil was taken from the high-yield field cultivation layer, air-dried, sifted through 8 mm to remove stones and roots, mixed with fertilizer, and sown, and then loaded into polyethylene plastic pots with a diameter of 50 cm and a height of 45 cm, and each pot was filled with 40 kg of air-dried soil. It was then filled into polyethylene plastic pots with a diameter of 50 cm and a height of 45 cm, with each pot containing 40 kg of air-dried soil.

### 4.2. Methods

#### 4.2.1. Experimental Treatments

The experimental site is located at the greenhouse experimental base of the Spice and Beverage Research Institute, Chinese Academy of Tropical Agricultural Sciences. The greenhouse cultivation environment features ample sunlight and a constant temperature of 30 °C. The soil of the research site was sandy loam with the following nutrient contents: organic matter 16.73 g kg^−1^, total nitrogen 0.68 g kg^−1^, available nitrogen 113.16 mg kg^−1^, available phosphorus 14.21 mg kg^−1^, and available potassium 91.33 mg kg^−1^. After being collected and mixed, the soil was placed into a pot with a diameter of 40 cm to eliminate soil heterogeneity.

The experiment employed a split-plot design with three AMF inoculation treatments: (1) non-inoculated control (NAM); (2) inoculated with *Claroideoglomus etunicatum* (AMF (C.e)); and (3) inoculated with *Funneliformis mosseae* (AMF (F.m)). Each AMF treatment had three nitrogen applications as secondary treatments: (1) conventional fertilization (C), (2) 30% reduction (−30%N), and (3) 60% reduction (−60%N). Each treatment had five replicates, with five pots per replicate, totaling 225 pots. Each pot was simultaneously planted with one vanilla and one areca seedling, both exhibiting uniform growth.

For conventional fertilization of potted plants, organic fertilizer is applied at a rate of 50 g kg^−1^, while CH_4_N_2_O is applied at a rate of 20 g kg^−1^. A 30% reduction means applying organic fertilizer at a rate of 35 g kg^−1^ and CH_4_N_2_O at a rate of 14 g kg^−1^. A 60% reduction means applying organic fertilizer at a rate of 20 g kg^−1^ and CH_4_N_2_O at a rate of 8 g kg^−1^. Apply foliar fertilizer 1–2 times per month. Water thoroughly every 3 days to maintain soil moisture at 70% of field capacity. Before transplanting seedlings into pots, gently comb through the root system and trim away any rotten, blackened, or excessively long damaged roots. Then disinfect the root system by soaking it in a low-concentration broad-spectrum fungicide solution (800–1000 times dilution of methyl thiophanate) for 5–10 min. After soaking, gently rinse the roots with clean water to remove residual solution before transplanting into pots containing 40 kg of air-dried soil that has been sterilized with a 0.3% potassium permanganate solution.

One month after transplanting, apply 5 g of fungal inoculant per pot to the two treatments requiring inoculation (AMF (C.e) and AMF (F.m)), while applying an equal amount of sterilized fungicide to the uninoculated control group (NAM). Dig small holes or trenches around the seedling roots along the root margins. The holes were dug 5–15 cm from the main stem, reaching a depth of 10 cm to ensure contact with the active root zone. The inoculant was evenly distributed into these holes, which were then filled with original soil and gently compacted to maintain soil moisture and prevent inoculant loss. After application, water all potted plants moderately with equal irrigation volumes to aid dispersal of the inoculant and facilitate contact with roots, promoting mycelial growth and root colonization. Samples were collected three months after fertilization treatment. Five potted plants were randomly selected from each treatment. Old and diseased leaves were removed from the vanilla and areca plants in each pot. The remaining leaves and roots were washed and drained. A portion of the roots was used for root activity determination, while the other part was directly frozen in liquid nitrogen and stored in a −80 °C freezer.

#### 4.2.2. Measured Indicators and Methods

##### Chlorophyll Content Determination

A handheld SPAD-502 Plus was used to randomly select the penultimate 5–6 mature leaves on the sunny side of areca and vanilla. Three leaves were taken, with three replicates, and the average value was calculated as SPAD value.

##### Photosynthetic Characteristics Determination

In the sunny and cloudless weather conditions of 9: 00 to 11: 00 on 25–29 September, the penultimate 5 to 6 mature leaves of areca and vanilla were randomly selected, and the net photosynthesis (P_n_), transpiration rate (T_r_), stomatal conductance (G_s_), intercellular CO_2_ concentration (C_i_), and other photosynthetic characteristics were measured by the LI-6400 portable photosynthetic instrument (LI-COR Company, USA). The photosynthetically active radiation was set at 1000 μmol m^−2^ s^−1^, and the carbon dioxide cylinder was used for gas supply. For specific methods, refer to the reference methods by Riches et al. [29,30]. Three leaves were randomly selected from each treatment for determination. After the data were stable, 5 times of data were recorded for analysis.

##### Determination of Soluble Sugar Content

The anthrone colorimetric method was used. Randomly select healthy mature leaves (the last 5–6 leaves) from vanilla and areca plants in each treatment for drying, then grind them into a fine powder. Accurately weigh a specific amount of the powdered sample, add 80% ethanol solution, and heat extract in a 60–80 °C water bath for 30 min to several hours. Add 15 g of activated charcoal for decolorization. After thorough shaking, centrifuge at high speed (10,000 rpm, 10 min) and collect the supernatant. Combine supernatants and evaporate in a water bath to remove ethanol. Concentrate to a specific volume, transfer to a volumetric flask, and dilute to mark with distilled water. Prepare glucose standard solutions (concentrations ranging from 7.0 to 42 μg/mL). Add anthrone reagent and concentrated sulfuric acid, mix thoroughly, and heat in a boiling water bath for 10 min. After reaction completion, rapidly cool to room temperature and measure absorbance at 630 nm using a spectrophotometer. Plot a standard curve with glucose concentration on the *x*-axis and absorbance on the *y*-axis. Take an appropriate amount of sample extract, add anthrone reagent and concentrated sulfuric acid, and react under the same conditions as the standard curve preparation. After reaction completion, cool to room temperature and measure the absorbance at 630 nm using a spectrophotometer. Determine the corresponding sugar concentration from the standard curve based on the measured absorbance value. Calculate the soluble sugar content in the sample by combining the sample dilution factor and sample mass, specifically, following the method described by Zhang Youjie et al. [31,32]:

(1) The soluble sugar content = [(amount of sugar obtained from the standard curve (μg) × extraction volume (mL) × dilution factor)/(volume of sample solution for determination (mL) × sample weight (g) × 10^6^)] × 100%

##### Determination of Soluble Protein Content

Coomassie brilliant blue staining method. Dissolve 100 mg of Coomassie Brilliant Blue G-250 in 50 mL of 95% ethanol. Add 100 mL of 85% phosphoric acid, dilute with water to 1 L, filter through filter paper, and store in a brown bottle for later use. Take out portions of plant leaves preserved in liquid nitrogen, grind them into a fine powder using a mortar and pestle, then transfer the powder to pre-chilled centrifuge tubes. Add an appropriate amount of protein extraction buffer PBS (phosphate-buffered saline). Mix thoroughly, then homogenize or shake in an ice bath for 30 min to 1 h to promote protein solubilization. Centrifuge at high speed (10,000 rpm) at 4 °C for 15–30 min. The supernatant constitutes the crude protein extract. Plot a standard curve using bovine serum albumin (BSA) as the standard protein and determine protein content via spectrophotometry. Detailed procedures refer to the methods of Wandan et al. [33,34], with the calculation formula as follows:

(2) Soluble protein content = C × V × dilution factor/W

In this context, C is the protein concentration in the sample solution as determined from the standard curve, V is the volume of the sample solution, and W is the weight of the sample.

##### Determination of Glutamine Synthetase (GS) Activity

Glutamine synthetase activity was assayed using a spectrophotometric method. The pretreatment of vanilla and betel leaf samples follows the same procedure as the determination of soluble protein content. Add glycerol as a protective agent, followed by Tris-HCl buffer at a specific pH, and protease inhibitors. Then add an appropriate volume of prechilled extraction buffer. After thorough mixing, the mixture was stirred or shaken at 4 °C for 30 min to 1 h to ensure complete protein solubilization. Subsequently, the mixture was centrifuged at high speed at 4 °C for 15–30 min. The precipitate was discarded, and the supernatant collected yielded the crude enzyme extract for subsequent protein quantification and glutamine synthase (GS) activity assays. Further optimize reaction conditions. After the reaction, add ferric chloride reagent to induce a colorimetric reaction with the generated γ-glutamylhydroxyoxamic acid. Measure absorbance at 540 nm. Calculate γ-glutamylhydroxyoxamic acid concentration using a standard curve and further estimate GS activity. Detailed procedures refer to the methods of Peng et al. [35,36], with the calculation formula as follows:

(3) GS activity (U mg^−1^ protein h^−1^) = ΔA_540_ × V_t_ (mL)/[ε (mM^−1^ cm^−1^) × d(cm) × V_enzyme(mL) × t (h) × C_protein(mg mL^−1^)]

In this context, ΔA540: the absorbance difference between the sample and the blank at 540 nm; Vₜ: the total volume of the reaction after addition of the terminating solution; ε: the molar absorption coefficient of the γ-glutamyl hydroxamate–iron complex; d: the path length of the cuvette; V_enzyme: the volume of crude enzyme solution (the volume added to the reaction); C_protein: the concentration of soluble protein in the crude enzyme solution.

##### Determination of Root Activity

Root vitality was determined using the tris(phenyl) tetrazolium chloride (TTC) reduction method. Root samples from vanilla and areca plants were cut into approximately 1 cm segments, and 0.5 g of each sample was accurately weighed into test tubes. Sequentially add 5 mL of 0.4% TTC solution and 5 mL of phosphate buffer (pH 7.0). Incubate in the dark at 37 °C for 2 h. Subsequently, add 2 mL of 1 mol/L sulfuric acid to terminate the reaction. Remove the root samples and extract them three times with ethyl acetate, combining the extracts. Measure the absorbance at 485 nm and calculate the TTC reduction amount based on the standard curve. This reduction amount represents root activity [37]. The TTC reduction intensity was calculated using the following formula:

(4) TTC reduction intensity = TTC reduction amount (mg)/root weight (g) × time (h)

##### Determination of Plant Nitrate Reductase (NR) Activity

The enzyme facilitates the reduction of nitrate to nitrite, and the accumulation of NO_2_^−^ in the solution is measured. The increase in NO_2_^−^ concentration in the reaction mixture indicates the enzyme’s activity. The determination of nitrate reductase activity was performed according to the method of Lea et al. [38]. Take 1 g of leaf sample and add 4 mL of pre-chilled extraction medium containing 0.1 M HEPES-KOH (pH 7.5), 3% (*w*/*v*) polyvinylpyrrolidone, 1 mM EDTA, and 7 mM cysteine. Grind on ice to prepare crude enzyme extract. Transfer 0.4 mL of the crude enzyme extract to a 10 mL test tube. Add 1.2 mL of phosphate buffer (pH 7.5) containing 0.1 M KNO_3_ and 0.4 mL of 2 mg/mL NADH solution. To distinguish total enzyme activity from actual enzyme activity, add either 2 mM EDTA or 6 mM MgCl_2_ to the reaction system. Incubate the mixture at 25 °C for 30 min. For the control tube, replace the NADH solution with an equal volume of 0.1 M phosphate buffer (pH 7.5). Immediately after reaction completion, add 1 mL of 1% (*w*/*v*) sulfanilamide solution to terminate the reaction. Subsequently, add 1 mL of 0.2% (*w*/*v*) α-naphthylamine solution and allow the color reaction to develop for 15 min. Centrifuge at 4000 r/min for 5 min, then measure the absorbance of the supernatant at 540 nm. Calculate the nitrite nitrogen content based on the sodium nitrite standard curve, and further determine the nitrate reductase activity per unit fresh weight of sample.

(5) Nitrate reductase activity = total nitrite nitrogen (μg) × buffer volume (mL)/fresh weight of sample (g) × enzyme solution volume (mL) × reaction time (h)

##### Determination of Acid Phosphatase Ctivity in Soil

p-Nitrophenyl phosphate method. p-Nitrophenol is hydrolyzed from p-nitrophenyl phosphate by soil acid phosphatase. The quantity of p-nitrophenol produced is measured using spectrophotometry. For each treatment, three soil samples were randomly selected. Using a 105 °C oven, the soil samples were dried to constant weight to determine soil moisture content. Soil pH was measured using a 2.5:1 soil-to-water ratio and a glass electrode pH meter (STARTER 300, OHAUS, USA). Soil available phosphorus content was determined by extraction with M3 solution (1:10 soil-to-water ratio) and analysis using a continuous flow analyzer (Skalar San++, city, The Netherlands). Soil acid phosphatase activity was measured using a microplate reader. The assay employed sodium acetate buffer (pH 5) as the buffer solution and p-nitrophenyl phosphate tetrahydrate as the substrate, incubated for 0.5–2.0 h [39,40].

(6) Acid phosphatase activity = concentration of p-nitrophenol (μg mL^−1^)× total extraction volume (mL) × dilution factor/sample mass × reaction time (h)

#### 4.2.3. Data Processing and Statistical Analysis

Using nitrogen application and *Arbuscular mycorrhizal fungi* inoculation as fixed factors, and blocks as random factors, a two-factor variance analysis was conducted to compare the effects of different treatments on soluble sugar content, soluble protein content, glutaminase, root, nitrate reductase, and acid phosphatase activity of vanilla and areca. Graphs were generated using Origin 2021. The data were analyzed using a one-way analysis of variation (ANOVA) for different cultivated patterns, including photosynthetic characteristics, soluble sugar content, soluble protein content, glutaminase, root, nitrate reductase, and acid phosphatase activity of vanilla and areca under different treatments. Data analysis was performed by using the Duncan test; the difference between mean values was determined by using the least significant difference (LSD, *p* < 0.05) as indicated by different letters. Before the data analysis, all the data whose input pattern was in accordance with the normality (Shapiro–Wilk) test. The correlations between the soluble protein content, root activity, and net photosynthesis were determined by Spearman’s correlation analysis. All data processing was conducted using SAS V8 software. The graphs were plotted by using Origin 2021.

## 5. Conclusions

The results of this study indicate that reduced nitrogen fertilization and AMF inoculation treatments significantly improved the photosynthetic characteristics, soluble sugar and protein content, root activity, and related enzyme activities of *Areca catechu* and *Vanilla planifolia*. In particular, inoculation with AMF, especially *Funneliformis mosseae*, significantly enhanced the photosynthetic efficiency of *Vanilla planifolia* and the nitrogen uptake efficiency of *Areca catechu*, mitigating the negative effects of reduced fertilization on the growth of both crops. The findings of this study will provide an optimized strategy for reducing chemical fertilizer application in tropical intercropping systems.

## Figures and Tables

**Table 1:** The average values of nitrogen fertilizer reduction and AMF inoculation synergistic regulation on vanilla photosynthetic characteristics. NAM, non-inoculated control; (AMF (C.e)), inoculated with *Claroideoglomus etunicatum*;(AMF (F.m)), inoculated with *Funneliformis mosseae*; C, conventional fertilization; −30%N, 30% reduction; −60%N, 60% reduction. SPAD value, chlorophyll content; P_n_, net photosynthesis; T_r_, transpiration rate; C_i_, intercellular CO_2_ concentration; G_s_, stomatal conductance. Data are presented as the mean ± standard error (n = 5). Values followed by different lowercase letters indicate significant differences at the 5% probability level (*p* < 0.05). Different capital letters (A, B, C) indicate significant differences among inoculation treatments at the same nitrogen application rate (*p* < 0.05).

Treatments	Fertilization Level	SPAD Value (mg g^−1^)	P_n_ (μmol m^−2^ s^−1^)	T_r_ (mmol m^−2^ s^−1^)	C_i_ (μmol mol^−1^)	G_s_ (mmol m^−2^ s^−1^)
NAM	C	27.07 ± 3.04 Aa	7.05 ± 0.85 ABb	0.53 ± 0.07 Aa	1878.02 ± 18.12 Bbc	0.005 ± 0.001 Bc
	−30%N	15.27 ± 2.85 Ab	2.52 ± 0.79 Bd	0.31 ± 0.02 Bbc	192.78 ± 8.78 Cc	0.010 ± 0.003 Ab
	−60%N	13.85 ± 2.94 Abc	2.09 ± 0.46 Bd	0.28 ± 0.02 Bc	130.77 ± 5.97 Cc	0.008 ± 0.002 Bbc
AMF (C.e)	C	13.81 ± 3.17 Bbc	4.27 ± 0.26 Bc	0.35 ± 0.06 BCb	1571.65 ± 16.06 Cc	0.009 ± 0.001 ABbc
	−30%N	12.27 ± 2.44 Ac	7.54 ± 0.63 Ab	0.45 ± 0.06 Aab	2135.20 ± 15.17 Bbc	0.012 ± 0.003 Ab
	−60%N	11.05 ± 2.12 Ac	7.51 ± 0.77 Ab	0.37 ± 0.05 Ab	2663.64 ± 18.69 Bb	0.017 ± 0.003 Aa
AMF (F.m)	C	6.45 ± 1.55 Bd	7.51 ± 0.86 Ab	0.28 ± 0.01 Bc	2633.05 ± 12.28 Ab	0.007 ± 0.001 ABbc
	−30%N	6.20 ± 0.91 Bd	8.43 ± 0.97 Aa	0.49 ± 0.03 Aa	2736.51 ± 13.91 Ab	0.012 ± 0.002 Ab
	−60%N	5.54 ± 0.90 Be	7.74 ± 0.76 Ab	0.36 ± 0.03 Ab	3274.11 ± 14.93 Aa	0.015 ± 0.002 Aa

**Table 2:** The average values of nitrogen fertilizer reduction and AMF inoculation synergistic regulation on the photosynthetic characteristics of betel nut. NAM, non-inoculated control; (AMF (C.e)), inoculated with *Claroideoglomus etunicatum*;(AMF (F.m)), inoculated with *Funneliformis mosseae*; C, conventional fertilization; −30%N, 30% reduction; −60%N, 60% reduction. SPAD value, chlorophyll content; P_n_, net photosynthesis; T_r_, transpiration rate; C_i_, intercellular CO_2_ concentration; G_s_, stomatal conductance. Data are presented as the mean ± standard error (n = 5). Values followed by different lowercase letters indicate significant differences at the 5% probability level (*p* < 0.05). Different capital letters (A, B, C) indicate significant differences among inoculation treatments at the same nitrogen application rate (*p* < 0.05).

Treatments	Fertilization Level	SPAD Value (mg g^−1^)	P_n_ (μmol m^−2^ s^−1^)	T_r_ (mmol m^−2^ s^−1^)	C_i_ (μmol mol^−1^)	G_s_ (mmol m^−2^ s^−1^)
NAM	C	28.39 ± 2.06 Bb	6.58 ± 1.16 Aa	0.22 ± 0.06 Bb	1581.63 ± 24.10 Acd	0.007 ± 0.001 Bc
	−30%N	15.96 ± 2.56 Bcd	1.25 ± 0.13 Cd	0.30 ± 0.03 Aab	193.91 ± 3.79 Cf	0.006 ± 0.001 Bc
	−60%N	14.86 ± 3.79 Bd	1.29 ± 0.09 Cd	0.16 ± 0.03 Ac	160.61 ± 5.21 Cf	0.005 ± 0.001 Bc
AMF (C.e)	C	36.17 ± 3.82 Aa	2.96 ± 0.21 Bc	0.19 ± 0.02 Bbc	617.81 ± 12.69 Ce	0.010 ± 0.002 Ab
	−30%N	29.11 ± 3.41 Ab	5.61 ± 0.24 Ab	0.31 ± 0.04 Aab	1428.59 ± 21.15 Bd	0.016 ± 0.002 Aab
	−60%N	20.89 ± 2.21 Ac	5.46 ± 0.37 Bb	0.05 ± 0.01 Bd	1884.95 ± 31.00 Bb	0.019 ± 0.002 Aa
AMF (F.m)	C	19.18 ± 2.90 Cc	6.36 ± 0.34 Aa	0.38 ± 0.03 Aa	1333.88 ± 14.85 Bde	0.010 ± 0.001 Ab
	−30%N	16.78 ± 2.91 Bcd	5.32 ± 0.43 Bb	0.27 ± 0.05 Ab	1682.97 ± 12.94 Ac	0.018 ± 0.003 Aa
	−60%N	14.55 ± 1.43 Bd	6.16 ± 0.53 Aab	0.16 ± 0.02 AAc	3179.21 ± 22.99 a	0.019 ± 0.002 Aa

**Table 3:** Effect of fertilization (N) and inoculation (A) and their potential interaction (N × A) on soluble sugar content in vanilla and areca.

Measurement Indicators	Treatments	Areca	Vanilla
soluble sugar content	N	546.64 ***	466.99 ***
	A	23.02 **	94.71 ***
	N × A	12.18 ***	152.22 ***

**Note:** N, fertilization; A, inoculation; N × A, fertilization and inoculation; Significant level: “**” indicate *p* < 0.01; “***” indicate *p* < 0.001.

**Table 4:** Effect of fertilization (N) and inoculation (A) and their potential interaction (N × A) on soluble protein content in vanilla and areca.

Measurement Indicators	Treatments	Areca	Vanilla
soluble protein content	N	0.41	2.02
	A	2.66 ^	4.05 *
	N × A	6.70 **	1.97

**Note:** N, fertilization; A, inoculation; N × A, fertilization and inoculation; Significant level: “^” indicate *p* < 0.1; “*” indicate *p* < 0.05; “**” indicate *p* < 0.01.

**Table 5:** Effect of fertilization (N) and inoculation (A) and their potential interaction (N × A) on glutamine synthetase activity of vanilla and areca.

Measurement Indicators	Treatments	Areca	Vanilla
GS activity	N	3.17 ^	4.65 *
	A	1.05	0.98
	N × A	0.59	1.32

**Note:** N, fertilization; A, inoculation; N × A, fertilization and inoculation; GS, glutamine synthetase. Significant level: “^” indicate *p* < 0.1; “*” indicate *p* < 0.05.

**Table 6:** Effect of fertilization (N) and inoculation (A) and their potential interaction (N × A) on root activity of vanilla and areca.

Measurement Indicators	Treatments	Areca	Vanilla
root activity	N	2.73 ^	1.03
	A	16.09 ***	20.84 ***
	N × A	2.91 *	3.81

**Note:** N, fertilization; A, inoculation; N × A, fertilization and inoculation; Significant level: “^” indicate *p* < 0.1; “*” indicate *p* < 0.05; “***” indicate *p* < 0.001.

**Table 7:** Effect of fertilization (N) and inoculation (A) and their potential interaction (N × A) on nitrate reductase activity in vanilla and areca.

Measurement Indicators	Treatments	Areca	Vanilla
NR activity	N	0.72	0.55
	A	1.28	0.75
	N × A	2.24	2.98 *

**Note:** N, fertilization; A, inoculation; N × A, fertilization and inoculation;NR, nitrate reductase. Significant level: “*” indicate *p* < 0.05.

**Table 8:** Effect of fertilization (N) and inoculation (A) and their potential interaction (N × A) on acid phosphatase activity in vanilla and areca.

Measurement Indicators	Treatments	Areca	Vanilla
ACP activity	N	3.67 *	8.34 **
	A	2.74 ^	6.31 **
	N × A	1.08	2.62 ^

**Note:** N, fertilization; A, inoculation; N × A, fertilization and inoculation;ACP, acid phosphatase. Significant level: “^” indicate *p* < 0.1; “*” indicate *p* < 0.05; “**” indicate *p* < 0.01.

## Data Availability

The datasets presented in this study can be found in online repositories. The names of the repository/repositories and accession number(s) can be found below: Mendeley Data, V1, doi:10.17632/f964jppzbv.1.

## Abbreviations

The following abbreviations are used in this manuscript:

AMF	Arbuscular mycorrhizal fung
NAM	non-inoculated control
AMF (C.e)	Claroideoglomus etunicatum
AMF (F.m)	Funneliformis mosseae
C	conventional fertilization
−30%N	30% reduction
−60%N	60% reduction
Pn	net photosynthesis
Tr	transpiration rate
Gs	stomatal conductance
Ci	intercellular CO2 concentration
GS	glutamine synthetase
NR	nitrate reductase
ACP	acid phosphatase
TTC	2,3,5-triphenyltetrazolium chloride
